# Self-Reported and Network Scale-Up Estimates of Substance Use Prevalence among University Students in Kerman, Iran

**Published:** 2018-04-30

**Authors:** Razieh Zahedi, Alireza Noroozi, Ahmad Hajebi, Ali Akbar Haghdoost, Mohammad Reza Baneshi, Hamid Sharifi, Ali Mirzazadeh

**Affiliations:** ^1^HIV/STI Surveillance Research Center and WHO Collaborating Center for HIV Surveillance, Institute for Futures Studies in Health, Kerman University of Medical Sciences, Kerman, Iran; ^2^Iranian National Center for Addiction Studies (INCAS), Tehran University of Medical Sciences (TUMS). Tehran, Iran; ^3^Research Center for Addiction & Risky Behaviors (ReCARB), Psychiatric Department, Iran University of Medical Sciences, Tehran, Iran; ^4^Modeling in Health Research Center, Institute for Futures Studies in Health, Kerman University of Medical Sciences, Kerman, Iran; ^5^Department of Epidemiology & Biostatistics, University of California San Francisco, San Francisco, CA, USA; ^6^Global Health Sciences, University of California, San Francisco, CA, USA

**Keywords:** Prevalence, Visibility, Substance use, University students, Network scale up

## Abstract

**Background:** This study aimed to estimate the prevalence of substance use among university students measured by direct and indirect methods, and to calculate the visibility factor (VF) defined as ratio of indirect to direct estimates of substance use prevalence.

**Study design:** A cross-sectional study.

**Methods:** Using a multistage non-random sampling approach, we recruited 2157 students from three universities in Kerman, Iran, in 2016. We collected data on substance use by individual face-to-face interview using direct (i.e. self-report of their own behaviors) and indirect (NSU: Network scale up) methods. All estimates from direct and indirect methods were weighted based on inverse probability weight of sampling university.

**Results:** The response rate was 83.6%. The last year prevalence of water pipe, alcohol, and cigarettes indirect method was 44.6%, 18.1%, and 13.2% respectively. Corresponding figures in NSU analysis were 36.4%, 18.2%, and 16.5% respectively. In the female population, VF for all types of substance was less than male.

**Conclusions:** Considerable numbers of university students used substances like a water pipe, alcohol, and cigarettes. NSU seems a promising method, especially among male students. Among female students, direct method provided more reliable results mainly due to transmission and prestige biases.

## Introduction

Substance use and its disorders have shown an increasing trend on the burden of diseases during the last decade^1^. This has a negative impact on several domains of life including familial, as well as occupational functioning^2^. Increasing trend of substance use was especially evident among adolescents and young adults in developing countries^3^. University students are particularly vulnerable to substance use ^4^ and it is associated with adverse effects including poor academic performance ^5^, higher risk of involvement in illegal activities, and being victim of rape and increased risk of premature death at young age ^6-9^.

Prevalence studies in universities of Iran, during 2002 to 2013, reported statistics which are of remarkably different. These differences might be justified partially as studies have been among different subgroups of university students in different times, settings, using different instruments and methods. Existing data reported lifetime prevalence of alcohol, water pipe, cigarettes and opium use among university students in the range of 11.8%-20%, 25.7%-40.3%, 18%-30.8% and 2.2%-10%, respectively^10-13^.

Direct methods are prone to different biases and usually lead to underestimation of true size ^14^. This is the case in particular when data are collected through face-to-face interview, instead of self-administered questionnaire. Network Scale-up (NSU) is an indirect method in which respondents provide us with number of their alters who engaged in risky behaviors in their network ^14^. On the other hand, the Achill hill of NSU method is visibility. This means that how much respondents are aware of the behavior of their alters.

Therefore, this study aimed to estimate the last year prevalence of substance use in the university students by direct and NSU methods, and to provide estimation for visibility factor (VF) defined as the ratio of indirect to direct estimates of substance use prevalence.

## Methods

### Study design and study population

We conducted a cross-sectional survey of 2157 university students, 1307 males, and 850 females, in three main universities (one medical, two non-medicals) in Kerman City, southeastern of Iran, in 2016. The Kerman University of Medical Sciences (KUMS), which is under supervision of the Ministry of Health and Medical Education, includes all medical and paramedical, and health-related disciplines at undergraduate and graduate level. The Shahid-Bahonar University, affiliated to the Ministry of Science and Technology, trains students, at under and postgraduate level, in engineering, social and basic sciences, and veterinary medicine. Shahid-Chamran also affiliated to the Ministry of Science and Technology, only enrolls males students at undergraduate engineering fields.

The Ethics Committee of KUMS reviewed and approved the study design and all procedures (IR.KMU.REC.1393.163).

### Eligibility and Sampling method

Students for at least one year were eligible to participate in the study. We used a quota sampling method. Sample size at each university was about 720. Following convince sampling and proportional to size approach, students were recruited from all departments. Sample size was calculated according to the prevalence use of alcohol reported 8.1%^15^. Assuming *P* to be 0.081, and d to be 0.013 we arrived at a sample size of 1693 at 95% significance level. Considering the refusal rate, we increased the sample size by about 20% and arrived at a maximum of 2032.

### Data collection

To decrease bias in answering to questions, trained interviewers explained the objective of the study to participants and ensured them on the confidentiality and anonymity of data. In the first section of the questionnaire, we directly asked students whether they had used any of substances even once in the past year. Data were obtained by means of a self-administered questionnaire and forms were dropped in a box.

In the second section, data were collected by NSU questionnaire, this method asks about the substance use in the respondent’s close friends. A trained same-sex interviewer asked students about how many of their close friends they know, used substance X at least once in the last year. Close friend was defined as ‘a university student that respondent knew him or her by name and face and contact with them several times a week and spent time with them at least two hours a week outside of the class’ ^16^. NSU questionnaire was completed by interviewers in a private place at the university. Based on the average number of their close friends that respondents know who use substance and the average personal network size (total of close friends), the proportion of students who use substance was estimated.

### Data management and analysis

The questionnaire was excluded in the case the respondents answered the initiation age of substance use questions but had negative reply to substance use. We adjusted for the clustering effect of universities in the analysis using survey analysis. All estimates were weighted based on the inverse probability weight of collage sampling.

Ratio of NSU over direct estimates was considered as a surrogate to Visibility Factor (VF). The visibility factors show the transmission of substance use behavior in close friends network in college students.

We used the chi-squared test for analysis of categorical variables and *t* -test for comparing two proportions. All of the statistical analysis done by Excel and Stata software, ver. 14 and significance level was in the 95% confidence interval.

## Results

Out of 2157 students, 1803 participated in the study giving response rate of 83.6% (78.8% in female and 82.9% in male students). In addition, 4% of the questionnaires were excluded due to unreliable replies (73 out of 1803). Final sample comprised of 1035 male and 695 female students (n=1730).

The mean (SD) age of the participants was 20.5 (1.5), with range of 18-29 yr. Mean age of male and female students were 20.4 (1.3) and 20.8 (1.6), respectively. Mean number of close friends for male and female student were the same (4.7 vs. 4.8, *P* =0.575). Majority of participants were single (98.9% male and 82.9% female, *P* <0.001). The age of traditional substance use initiation in male was lower than female (15.3 ±0.10 versus 16.9 ±0.4). Corresponding figures in terms of industrial drugs were 15.2 ±0.3 and 17.7 ± 0.4). Age of alcohol use initiation in male was 15.4(0.2) and female 18.2(SD 2.8), (*P* <0.001 in all cases).

### Direct estimates

The most prevalent substances among male students, and female students, were water pipe (53.4% versus 31.9%, *P* =0.006); tramadol, diphenoxylate, or codeine (26.5% versus 38.6%, *P* <0.001); alcohol (23.7% versus 9.7%, *P* <0.001); and cigarettes (16% versus 9.1%, *P* <0.001) during last year.

In both gender the reported last year prevalence of heroin, methamphetamine, and chewing tobacco was less than 1%. The prevalence of opium use was less than 1% only in female sample (Table 1).

### NSU estimates

Results of NSU analysis were the same as direct in terms of substances which were more prevalent. The only difference was that prevalence of tramadol, diphenoxylate, or codeine in female students was higher (38.6% in male and 51.01% in female, *P* <0.001) (Table 1).

**Table 1:** The prevalence of substance use in last year among university students by type of substances, sex and measurement methods, Kerman, Iran 2015

**Substance**	**Male (n=1035)**			**Female (n=695)**			**Total (n=1730)**		
	**Direct%** **(95% CI)**	**Indirect%** **(95% CI)**	**Visibility** **coefficient** **(** ***P*** **value)** ^a^	**Direct%** **(95% CI)**	**Indirect%** **(95% CI)**	**Visibility** **coefficient** **(** ***P*** **value)** ^a^	**Direct%** **(95% CI)**	**Indirect%** **(95% CI)**	**Visibility** **coefficient** **(** ***P*** **-value )** ^a^
Cigarettes	16 (13.4, 18.6)	24.9 (22.1, 27.8)	1.6 (0.001)	9.1 (6.2, 12.1)	3.4 (2.1, 4.7)	0.4 (0.001)	13.2 (11.3, 15.2)	16.5 (14.4, 18.6)	1.3 (0.001)
Water pipe	53.4 (49.9, 56.9)	50.8 (47.6, 53.9)	0.95 (0.127)	31.9 (27.5, 35.6)	13.4 (10.7, 16.2)	0.4 (0.001)	44.6 (41.9, 47.4)	36.4 (33.8, 39.0)	0.8 (0.001)
Pipe	5.5 (3.8, 7.2)	2.3 (1.7, 2.9)	0.4 (0.001)	1.4 (0.2, 2.6)	0.06 (0.0, 0.2)	0.04 (0.026)	3.8 (2.8, 4.9)	1.4 (1,1.8)	0.4 (0.001)
Chewing tobacco	0.6 (0.1, 1.1)	1.0 (0.7, 1.4)	1.7 (0.222)	No data	0.06 (0.05, 0.2)	No data	0.3 (0.05, 0.6)	0.6 (0.4, 0.9)	2.0 (0.124)
Opium	2.1 (1.1, 3.1)	1.8 (1.1, 2.4)	0.9 (0.515)	No data	0.07 (0.05, 0.2)	No data	1.2 (0.6, 1.8)	1.1 (0.7, 1.5)	0.9 (0.719)
Heroin	0.07 (0.02, 0.2)	No data	0.0 (0.062)	No data	No data	No data	0.04 (0.02, 0.1)	No data	0.0 (0.069)
Cannabis	4.3 (2.9, 5.7)	6.2 (4.7, 7.6)	1.4 (0.018)	2.7 (0.3,0.8)	0.9 (0.02, 1.7)	0.3 (0.001)	2.7 (1.8, 3.5)	4.1 (3.1, 5.1)	1.5 (0.006)
Methamphetamine	No data	0.1 (0.01, 0.2)	No data	0.3 (0.3, 0.8)	0.06 (0.05, 0.2)	0.2 (0.072)	0.1 (0.1, 0.3)	0.1 (0.02, 0.2)	1.0 (0.001)
Ritalin	1.7 (1.0, 2.3)	2.4 (1.8, 3.0)	1.4 (0.169)	17.8 (15.8, 19.9)	2.1 (1.1, 3.0)	0.1 (0.001)	8.2 (7.3,9)	2.3 (1.7, 2.8)	0.3 (0.001)
Tramadol, diphenoxylate or codeine	26.5 (23.4, 29.6)	38.6 (35.2, 42.0)	1.5 (0.001)	49.2 (44.6, 53.7)	51.01 (46.3, 55.7)	1.0 (0.388)	35.6 (33.01, 38.2)	43.3 (40.5, 46.2)	1.2 (0.001)
Sedative and hypnotic medications	6.0 (4.4, 7.6)	2.9 (2.1, 3.7)	0.5 (0.001)	26.2 (22.9, 29.5)	5.6 (4.01, 7.2)	0.2 (0.001)	14.5 (12.9, 16.2)	3.9 (3.1, 4.8)	0.3 (0.001)
Alcohol	23.7 (20.8, 26.8)	26.4 (23.6, 29.2)	1.1 (0.071)	9.7 (6.7, 12.7)	5.5 (3.9, 7.1)	0.6 (0.001)	18.1 (15.9, 20.3)	18.2 (16.2, 20.3)	1.0 (0.921)

^a^ Comparison indirect ratio to direct estimate method

In the male population, less prevalent substances by NSU was the same as that of direct method (heroin, methamphetamine, and chewing tobacco). In the female population, in addition to four substances selected by direct method, NSU estimates selected two more substances were less than 1% (pipe and cannabis) (Table1).

### Visibility Factor

In the female population, VF value for almost all types of substance was less than one. Estimates from NSU were lower than direct method. In male population, VF value for pipe and sedative and hypnotic prescriptions was remarkably lower than one (0.4 and 0.5). For three substances (cigarette, cannabis, and tramadol) VF value was remarkably higher than one and for two other substances (chewing tobacco and Ritalin) VF value was higher than one but it was not significant (Table 1).

## Discussion

Our results showed that more than one-third of university students reported waterpipe used in last year; about 18% reported using alcohol and also about 15% reported using cigarettes in last year. In comparison to female students, the prevalence of reported substance use was significantly higher among male students.

Methods of estimation significantly changed the results for Ritalin and sedative. VF for the former was higher than one among male but lower than this threshold among female students. VF for sedative was lower than one in both genders (0.5 in male and 0.2 in female students).

The high prevalence of water pipe (17.9% to 25.7 %)^10,17,19^, alcohol (7.9% to 11.8 %)^>10,17,19^ and cigarettes (12.4% to 18.0%)^10,17,20^ was also reported in the previous studies of university students in Iran. However, in Tunisian, alcohol, Tobacco, and cannabis were the most common substances of use. Alcohol consumption in this study compared other Islamic countries, in some case like Tanzania was higher ^21^, however in some cases was lower ^22,23^. Studies reported substance use vary widely depending on gender^24^, ethnic, religious, geographical and cultural differences ^25,26^. Over the last decade, studies reported an uprising trend of water pipe use which can be due to a lower perceived risk and stigma attached to water pipe use compared to cigarettes ^27^, so water pipe use is perceived to have lower risk and higher social acceptability among university students^28^.

Substance use in male was more than female students except for drugs such as Ritalin, tramadol diphenoxylate or codeine and sedative that was consistent with results of previous studies in Iran^29-31^ and other countries^11,32^. Some studies reported sedative consumption in women more than male ^33,34^.

While in the female population VF for most of the substances was less than 1, the opposite was true for male students. In the female population, direct estimates were higher than NSU estimates. Female students do not share their sensitive behaviors even with their close friends. If transmission of substance use in network of students was high it could lead to spread this behavior and fade the abomination of these behaviors in students. On the other hand, as for male students, NSU estimates were higher. Two hypotheses are that male students do not reply direct questions as honest as female students, or they exaggerate risk behaviors in their network.

Our study had some limitations. First, about 16% of the population refused to participate in our study. Second, we defined substance use as at least one episode of substance use in last year, which is different form disorder definition. Third, our estimates of substance use prevalence in direct and indirect method were subjected to sociality desirability bias, transmission error, barrier error and so estimates from these methods might have underestimated the true prevalence. Last, we studied substance use in university students, not in all youth population, and so generalizability of our findings is limited to the studied population.

## Conclusions

A considerable number of university students use substances like water pipe, alcohol and cigarettes recently. NSU seems a promising method, especially among male students. Among female students, direct method provided more reliable results mainly due to transmission and prestige biases. VF in male close friend’s network was more than female counterparts that suggest higher probability of substance use transmission in males students’ network compared to females. Regular interventions, to prevent students from starting substance use or shifting towards substance use disorders, are needed.

## Acknowledgements

The authors are grateful to HIV/STI Surveillance Research Center, Kerman University of Medical Sciences and Iranian National Center for Addiction Studies (INCAS), Tehran University of Medical Sciences for funding the research. We would like to thank Zeinali Masood, MSc, and interviewing team for assisting data collection.

## Conflict of interest statement

None of the authors have any financial or other interests that might influence the conduct of the study or accurate reporting of the results.

## Funding

This work was supported by Modeling in Health Research Center and HIV/STI Surveillance Research Center, and WHO Collaborating Center for HIV Surveillance, Institute for Futures Studies in Health, Kerman University of Medical Sciences, Iran in cooperation with the Communicable Diseases Center and Office of Mental Health, Social, and Addiction Department of the Ministry of Health and Medical Education.

## Highlights

- NSU method can be used as indirect size estimation.
- Water pipe use is very common in university students.
- Transmission of sensitive information in male students’ network is higher than girl students.
